# Fitness for all: how do non-disabled people respond to inclusive fitness centres?

**DOI:** 10.1186/s13102-021-00303-2

**Published:** 2021-07-30

**Authors:** Helene Nikolajsen, Emma Victoria Richardson, Louise Fleng Sandal, Birgit Juul-Kristensen, Jens Troelsen

**Affiliations:** 1 Research Unit for Musculoskeletal Function and Physiotherapy, Department of Sports Science and Clinical Biomechanics, University of Southern Denmark, 5230 Odense, Denmark; 2 Department of Physiotherapy, Institute of Health Studies, University College South Denmark, Esbjerg-Haderslev, Denmark; 3 School of Sport and Exercise Science, University of Worcester, Worcester, UK; 4 Research Unit for Physical Activity and Health in Work Life, Department of Sports Science and Clinical Biomechanics, University of Southern Denmark, Odense, Denmark; 5 Research Unit for Active Living, Department of Sports Science and Clinical Biomechanics, University of Southern Denmark, Odense, Denmark

**Keywords:** Qualitative research, Focus group interviews, Fitness, Fitness centre, Gym, Inclusive fitness centre, Disabilities, Inclusion

## Abstract

**Background:**

Representation of people with disabilities in fitness centres is lacking, despite initiatives to promote inclusion mainly in the UK and USA. Success creating these inclusive spaces is mixed and few were crafted taking into account attitudes and biases of non-disabled co-members. Inclusive fitness centres have not gained much attention in Denmark, and the campaign ‘Fitness for All - fitness for people with physical disabilities’ was initiated. The aim of this study was shaped by two key questions; 1) what is the ideal fitness space from the perception of non-disabled fitness users? and 2) how might their dis/ableist attitudes negate inclusion in three future pilot inclusive fitness centres across Denmark?

**Method:**

Three focus groups involving 5–7 (total *n* = 18) adult non-disabled participants were conducted. Aged ranged between 19 and 75 years, both men and women were involved, with fitness centre experiences ranging from 0 to 20+ years. Interviews were transcribed and analysed using Malterud’s four-step method of systematic text condensation.

**Results:**

Of most importance was a pleasant atmosphere which should make them feel welcome and comfortable. Good social relations within the space were also highly valued. Participants welcomed people with physical disabilities but predicted many challenges with an inclusive fitness centre and expressed unconscious ableist attitudes.

**Conclusion:**

The current study adds essential knowledge regarding how non-disabled people perceive the ideal inclusive fitness centre. A welcoming and inviting atmosphere is essential whereas social skills, ableism, ignorance, and preconceptions are important barriers that may hinder inclusion of participants with disabilities in inclusive fitness centres.

**Supplementary Information:**

The online version contains supplementary material available at 10.1186/s13102-021-00303-2.

## Background

Despite the global focus on physical activity and health and a booming fitness industry, there is a lack of people with disabilities in fitness centres [1, 2]. This is a considerable problem as about 15% of the world’s population is estimated to live with some form of disability [3] and this group has a higher prevalence of illness and lifestyle diseases related to inactivity [4]. As well as reduced health-related benefits and decreased physical function, psychological and social health can be impacted due to inactivity as this can enhance feelings of isolation, stigmatisation, and lack of social relations [5].

Fitness centre training appeals to a broad audience of people with disabilities because of relatively easy access, flexible hours and no requirements of specific physical skill (e.g. like ball games) or physical fitness level [6]. However, they are perceived as a particularly exclusive space by people with disabilities [7]. Using a critical disability studies lens and contesting conditions of ableism and disablism in society [8], numerous complex and interrelated phenomena illuminate why people with disabilities are excluded and underrepresented in fitness centres [9].

Ableism frames images, policy and discourses as if all people are non-disabled [10] excluding any representation of a different physical form [11]. Ableism values self-sufficiency, autonomy and independence, leading to the exclusion of many people who do not align to a culturally created imagery of ‘ableness’ [8]. Ableism is proposed as a regulator within sport and exercise settings, including fitness centres, as they are often spaces that value one particular muscular, fully functional, aesthetic physical form, leading to the exclusion of people with disabilities in these spaces [12]. This leaves people with disabilities feeling intimidated, unwelcome, excluded, and oppressed in this setting [13].

Disablism, which refers to the social oppression people with disabilities experience from the physical environment and relationships with others [14, 15], can be another barrier to inclusion in fitness centres. It arises in two different forms; (1) indirect and (2) direct psycho-emotional disablism. Both are keenly apparent within fitness centres. Indirect psycho-emotional disablism relates to structural barriers that exclude people with disabilities from physical spaces and project messages that this community is not welcome and does not belong [16]. Fitness centres send these exclusory messages through lack of physical access, inaccessible changing facilities, unsuitable equipment and lack of space to transfer to equipment [2, 17–19]. Direct psycho-emotional disablism refers to the negative interactions people with disabilities have with others such as negative or invalidating responses, being stared at, having jokes made about them, or dealing with callous remarks or comments which can result in feelings of anger, otherness, lacking self-worth and feeling excluded [20]. Both ableism and disablism are substantial barriers in fitness centres in UK [13, 21].

Over the last 2 decades, research has focused on identification of barriers and facilitators of disability inclusion in fitness settings (e.g. [1, 7]). This dearth of research shows that over this time, little has changed as the same structural, attitudinal, and relational issues such as no access, ableist and disablist interaction are continually reported [1, 2, 7, 13, 17, 22]. As a result, scholars have called for academics to move beyond these types of exploratory studies as, at this point, findings are merely repetitive and descriptive as the inclusion of people with disabilities has changed so little adopting this approach [23]. Instead, scholars must take the leap to develop strategies to address inclusion issues rather than merely describe them [22].

In this research, we move towards developing a strategy to improve inclusion in fitness centres and a country that has yet to be contextualised within the greater global disability inclusion movement. Disability research is strongly represented in welfare states in Scandinavia, however Denmark is lacking behind with fewer research environments than both Norway and Sweden [24]. Thus, Denmark requires particular attention for improving and promoting disability inclusion. In Denmark, as in other countries, people with disabilities have lower levels of education and fewer people are in the labour market [25, 26]. This makes leisure time and associated activities an opportune place for people with and without disabilities to meet [27], as such, fitness centres may be a meaningful place where disability prejudice can be broken-down. Unfortunately, leisure time sporting activities in Denmark are segregated into ‘traditional’ sport (non-disabled) and parasport which does not align to inclusion. As such, creating an inclusive fitness centre could be the first step to meet on equal terms and reduce prejudice. As there is little work on disability inclusion in Denmark, there is an exciting opportunity not only to promote inclusive sport and exercise, but also to create a space that is truly inclusive by addressing dis/ableist attitudes. To bring disability inclusion to attention, the campaign ‘Fitness for All- fitness for people with physical disabilities’ was initiated, establishing three new pilot inclusive fitness centres across Denmark. This programme sought to rethink non-profit, club-based fitness centres and create an equitable space for both people with and without disabilities as peers. An inclusive exercise space may not only provide a space for people with disabilities to access equitable fitness opportunities but also educate non-disabled people about disability and reduce ableist prejudice. Further, results from this research could begin the important dialogue of informing the design of a fully inclusive fitness centre that will satisfy both groups and inform other fitness centres in inclusive practice.

Thus, the purpose of this paper was to improve inclusion in fitness centres by first identifying the ableist attitudes we will inevitably encounter from non-disabled members. This underscored our aim of identifying ableist barriers to inclusion, wherein we could anticipate the potential barriers, attitudes and perceptions that may hinder inclusion, and address these before members with and without disabilities use this exercise space. The aim was shaped by two key questions; (1) what is the ideal fitness space from the perception of non-disabled fitness users? and (2) how might their dis/ableist attitudes negate inclusion in three future pilot inclusive fitness centres across Denmark?

## Methods

We adopted a qualitative, cross sectional design whereby we sought to develop an in-depth, detailed data set of Danish non-disabled persons’ perceptions of an inclusive fitness centre. The steering committee of the project ‘Fitness for all - fitness for people with physical disabilities’ selected the three specific centres for intervention after receiving applications from potential non-profit and club-based fitness centres to be a part of the campaign. The chosen non-profit, club-based fitness centres are located in three different municipalities in Denmark; one was located in a village awaiting an extension and establishment of a fitness centre, another was a small fitness centre awaiting a new and bigger building within an already established sports club in a minor city and l the third was a newly established fitness club awaiting a building were under reconstruction located in a suburb to a big city. A focus group interview was conducted at each location with a group of non-disabled adults. The interview project was scientifically approved by the University of Southern Denmark, Research and Innovation Organisation on behalf of The Danish Data Protection Agency, journal number 2015-57-0008. The COREQ checklist for qualitative interviews and focus groups [28], was used for reporting.

### Sampling and participants

Employees/volunteer workers at the three selected fitness centres acted as gatekeepers to participants and were partly responsible for recruiting the participants for the focus groups. They were asked to compile a list of ‘potential fitness users of the coming inclusive fitness centres’ with information about gender, age and fitness centre-experience (limited: none to a few visits in fitness centres, former: regular fitness users/membership in the past, and current: active user in a fitness centre at the time of the interviews). The list was used to secure maximal variation of the participants included in the study. This enabled a wide and in-depth range of experiences and demographics to be collected that would allow for comprehensive accounts of non-disabled persons perceptions of inclusive fitness centres.

The inclusion criteria for the participants defined as ‘potential members’ were; adults (≥ 18 years) who were users of the already established fitness centres and/or future users of the upcoming three inclusive fitness centres. Participants were excluded if they had physical or cognitive disabilities or a severe visual or hearing disability or were unable to speak and understand Danish. Participants’ previous experience and contact with people with physical disabilities were not taken into consideration in the sampling process.

Participants were recruited through a notice in the local fitness centre or through relevant groups on a social media platform supplemented with snowball recruitment. The gatekeepers used snowball recruitment in their network to compile the list of potential users. Further, we used snowball recruitment when contacting the persons on the list if we lacked participants of a specific gender or age, especially when recruiting the younger participants. In total, 18 people (nine females and nine males) participated in the interviews (see Table 1). The three focus groups comprised five-to-seven people each and participants were contacted by telephone by the first author to orally confirm their interest in participation, double check the eligibility and to secure maximal variation within groups in terms of gender, age and fitness centre experience. Fitness centre-experience was self-reported, and the information was validated through the information from the interviews. Further, the participants could ask questions and obtain more detailed information about the practical arrangements of the interview and the relation to the 'Fitness for all-campaign'. Written informed consent was obtained before the interviews. All names reported in this article are pseudonyms.

**Table 1:** The three focus group interviews; numbers of participants, gender, age, and fitness centre experience

Focus group interviews	Numbers (female/male)	Age range (years)	Mean age (years)	Fitness centre experience (limited/former/current)
Location 1	6 (3F/3M)	19–51	36	3/2/1
Location 2	7 (5F/2M)	23–75	55	1/5/1
Location 3	5 (1F/4M)	19–67	54	0/1/4
Total group	18 (9F/9M)	19–75	48.5	4/8/6

### Data collection

Data were collected using a focus group at each fitness centre. Focus groups were used as they facilitate the creation of new knowledge in areas that are underresearched, bring forth spontaneous, dynamic dialogue between people, participants have a higher degree of control over discussions, and people may be more willing to discuss things in depth as part of a group rather than one on one [29]. A semi-structured guide with open-ended questions (see Additional file 1, for an English version) was developed for this study to ensure both width and depth in the focus groups. To increase internal validity, two pilot interviews were conducted with 2 and 3 participants respectively, all were current fitness centre users in a similar non-profit club-based fitness centre setting but at another location. Only small adjustments were performed by adding extra cues to the interview guide and rephrasing a few questions to facilitate participant specific examples of their experiences.

The guide was developed with three overall themes: (1) the physical surroundings and accessibility, (2) activities and usability, and (3) atmosphere in the fitness centre. Broad open-ended questions were composed for each of the themes, focusing on the participants’ experiences and perceptions - both positive and negative. Examples of questions included: What are your experiences with fitness centres? What is good accessibility to you? Where do you experience problems? How do you use the fitness centre (both in the past, present and future)? How do we make a successful inclusive fitness centre for both people with and without physical disabilities? Pros and cons? All three themes were discussed in each interview, but the order differed and as the topics are linked together the conversation naturally jumped from one topic to another. Further, all subthemes were mentioned within each of the interviews.

The focus groups were conducted by the first author acting as moderator at the three different locations, which was a meeting room either in relation to the coming fitness centres in the local sports club or in the city hall. The interviewer (first author) has a background as a trained physiotherapist, MSc in Health Science and has personal experience with both non-profit sport clubs and commercial fitness centres. Therefore, she was familiar with the jargon in the interviews, but had no associations with the 3 fitness centres and no local knowledge. The interviews were conducted as a part of a PhD-study. Only the first author and the participants were present during the interviews. The duration of each interview was 98–112 min, which led to a total of 5 h and 10 min data material from all three focus group interviews. The interviews were conducted in March and April 2018. Field notes was made after each interview, to get capture new reflections after each interview.

### Data analysis

The audio recorded interviews were transcribed in a slightly modified verbatim mode as proposed by Malterud [30]. That is, focusing on the content of the interviews and carefully making smaller adjustments from spoken language to written language e.g., by erasing repetitions and empty words and adding punctuation. The first author performed the transcriptions. With a descriptive and explorative analytical approach, the data analysis was thematic with a cross-case approach and data driven. The analysis was performed in 4 steps, following the Systematic Text Condensation (STC) method by Malterud [31]. The four steps were: (1) total impression—from chaos to themes, (2) identifying and sorting meaning units—from themes to codes, (3) condensation—from code to meaning, and (4) synthesizing—from condensation to descriptions and concepts.

Four authors (HN, LFT, EVR, JT) were involved in the analysis, focusing on the participant´s perceptions on fitness centres, the non-profit club format, and the new inclusive concept. The coding was performed in Nvivo 12 software. An initial coding process (step 1) was performed by two researchers (HN and LFT) to ensure structure and content of the analysis. The first author performed the coding (step 2) and the overall analysis was performed with many different meaning units on a detailed level from the beginning and subsequently grouped together in code groups and subgroups. Two authors (HN and EVR) discussed the code groups and subgroups (step 3) and (HN and JT) discussed the analytical categories (step 4).

## Results

According to the analytical categories, the results was divided into two subsections. First, an account of how the participants describe their ideal fitness centre, and secondly perceptions of sharing an inclusive fitness centre with participants with physical disabilities. Interview no. 1, 2 and 3 refer to the three different locations for interview.

### The ideal fitness centre: room for comfort and diversity

#### Basic expectations for a non-profit club-based fitness centre

The participants had certain expectations for the upcoming inclusive Danish non-profit fitness centre. Locations with easy access both by car, bicycle or public transportation were highlighted as very important. If the location was considered inconvenient, they would not use it. Further, participants requested a bright, welcoming, and well-maintained, clean environment to make the exercise setting attractive and comfortable. Susanne explained:

I think it is important with light, how it falls and the illumination. Colours on the walls and not in the linoleums-municipality-way, and no smelly rubber. […] so, when you go in you think ‘this is a nice place to be’; I like to be here because something is calling for me. (Interview 2)

The participants stressed the discomfort of the stench of sweat and rubber, and the intimidation of posters and other media on the walls with ‘protein-commercials’ and extreme examples of ‘fit’ men and women. In general, the participants drew upon past experiences of a traditional commercial fitness centre. Marie-Louise talked about one of her experiences:

When I started exercising, I thought the easiest thing was to start in my local commercial fitness centre right across the street. I opened the door and there it was; the smell of sweat, the loud music going on ‘duff duff duff’ and the very high stress-level. So, I thought, this is not what I’m looking for. […] Later when I became a more experienced fitness centre user I went back to try again, but I am not going in there; it’s a no-go. (Interview 2)

Regarding the use of space, the participants underlined the importance of the ‘right’ training equipment and room for socialising. They also asked for flexibility. They expected long opening hours (e.g., from 5 am to 11 pm) with key tags so, members could come and go as preferred. The price level for membership and how to get value for money was very much debated among the participants. They sought a balance of price levels between very cheap prices in volunteer sports clubs and more expensive in commercial fitness clubs. Participants generally agreed price levels of 100–150 DKK (13–20 EURO) per month would be reasonable. Maya stressed:

If it costs 35 Euro a month, and if I only come once every two weeks, I must admit that I am too stingy for it. (Interview 1)

#### User exercise knowledge and skills are required

The participants all agreed that basic user competences were required to exercise in a fitness centre and stated that if someone did not know what to do and why, then they would never enter or be a regular user. Therefore, in order to feel comfortable (especially newcomers) the participants strongly recommend that an introduction session would be very important, e.g., one-on-one sessions or small group introductions. David talked about his practical limbo:

I would sit on such a machine and say, ‘uh yes, what next?’ I’ve been practicing handball and soccer, and like to run for a ball, but jumping on a treadmill…, I’ve never tried it, so I think I would fall off. (Interview 1)

Participants also found it very important to have someone to consult with regarding how to use the fitness equipment, compose/adjust exercise programs and someone to lead classes and maintain the equipment. They were aware of potentially heavy employee costs, so participants suggested volunteer instructors should be available on specific hours, or collaboration with educated professionals or students within sports science or physiotherapy. Marie-Louise told how it was done in her non-profit fitness centre:

The volunteer staff have to be users of the fitness centre, because they are often there anyway and know exactly how all the machines work so they can assist others […] Being a volunteer is only something you do if you gain something out of it. It could be free instructor courses, fitness clothes, paid membership and a dinner once a year with all the other volunteer staff. (Interview 2)

#### Rules and behaviour in fitness

The participants were very engaged regarding how to run codes of practice., i.e., etiquette, and rules regarding how users should behave and what is allowed in the centre (e.g., in relation to doping issues.) Several examples were brought up about annoying behaviour such as inconvenient use of equipment and mobile phones, inappropriate attire, failing to clean-up or forgetting to wipe off the fitness machines after use. Charlotte illustrated:

I get so annoyed if people sit on a machine or bench without exercising, then I say, ‘So, do you use it as an armchair or what?’ (Interview 3)

In general, participants wanted to confront other members in a polite, suitable, or humoristic way, but found it hard to do as an ordinary member and believed it was easier to do for the volunteer staff with more authority. Issues regarding other users who puffed and groaned aloud, sweated, smelled, or became noisy when using the equipment was considered harder to regulate. Birger gave an example of an uncomfortable situation:

Some time ago, a woman used to come and work out in the fitness centre, and not many liked her because she smelled. The other users knew when she used to come and exercise, and they simply stayed away or came an hour later. (Interview 3)

#### The atmosphere: fitting in with social relations

The participants kept returning to talk about atmosphere or the ‘right spirit’ in the fitness centre as a key aspect when deciding if they would actually use the fitness centre or not. They stated the importance of felling that they ‘fit in’. Sylvester explicated:

Many times, when you come into such a fitness centre, you feel so overlooked because you have such a feeling that it is a crowded bunch and the users come in such super smart clothes and everything. So, it must be a place that is nice to come and where you feel at home. (Interview 1)

The feeling of belonging and fitting in was perceived to be possible if there were greetings when seeing others, sharing the space with peers in terms of similar age, appearance, and preferences for specific training types. In particular, participants discussed intimidation of not being able to live up to super fit body norms with big muscles or skinny appearance, which made them feel uncomfortable, out of place and not welcome. Contrary to many commercial fitness centres, they wanted a place without body-shaming with room for ‘normal’ overweight persons. Tommy summed up:

This new fitness centre should be for everybody – Fitness for all – it has to address the local people, so as you say, there should not be any body-shaming – it should be a place for Mr. and Mrs. Denmark or Mrs [name of the town]. (Interview 1)

Social relations were also very important for participants. They noted enjoyment in meeting people they knew, but also making new acquaintances. Often new relations began with small talk, progressed to a cup of coffee and later developed into friendships. In general, participants found other users friendly and helpful. Tommy gave examples:

After all, most people are kind and sweet if you ask: ‘Sorry, can you please tell me how to do this?’ Or if they can see that it is completely hopeless what you are doing, then most people can also come and say: ‘Shouldn’t I just show you how to do this?’ or ‘Shouldn’t I just lend you a hand?’ (Interview 1)

Generally, participants expressed the need for good social relations for long-term commitment to exercise. Being part of a team who exercised regularly, had fun, and met in the cafeteria afterwards were noted as very important. Although some preferred to exercise on their own, the majority preferred training in smaller groups of 2–5 persons matched by age, fitness type and fitness level. The participants underlined the importance of social relations and being part of a club based on experiences from other sports clubs they had been members of earlier in life. Josefine gave an example:

If it is a club, then there should also be a common room where you can sit down and drink sodas and meet people and have the opportunity to talk. Otherwise, it’s not a club. (Interview 2)

### Ideal inclusive fitness centres: reflections on sharing a fitness space with people with disabilities

#### The degree of disability

All participants responded very positively towards establishing new inclusive fitness centres for both fitness users with and without disabilities. Several participants made clear that people with disabilities were more than welcome to join. However, there were also inherent ableist perceptions and statements made such as others may choose a different fitness centre because of the presence of people with disabilities and the further inclusion of people with disabilities should not happen at the expense of those people without disabilities who were already using the fitness centre.

Specifically, the participants were focused on the severity of a member’s disability., i.e. whether that person required a carer, could exercise independently or something in between. Ib was straightforward, but also showed some already inherently ableist perceptions of members with a disability:

You could be crude and say that when we say ‘disability’, we do not really mean the multi-disabled who need help with everything, right? It is the ones who – you can say – in many cases are self-sufficient, possibly supported by a carer. (Interview 3)

#### Adaption of settings

The participants quickly address the requirements for physically inclusive adjustments such as lifts, extra space for wheelchairs and zones with special fitness machines suitable for both people with and without disabilities. They also discussed the need for extra cleaning when dirty wheelchairs enter a centre where only indoor shoes are allowed. Several of the participants stated the importance of securing the feeling of a volunteer fitness centre with no resemblance to hospitals, rehabilitation centres or other medicalised buildings. Charlotte reflected on the sense of belonging:

I may be naive, but I think we can easily make a disability-friendly centre where people can get around and where things are placed so that it fits when sitting in a wheelchair, but still so that we others can be there without feeling we're in a hospital room. (Interview 3)

#### Social codex for inclusive centres

Participants discussed separated or integrated training classes but struggled on how to put this into practice. Tommy summed up:

I think it is harder to adjust so they can participate in our classes than for us to participate in disabled classes because of the big difference; we run, do push-ups and squats etc. It would be hard to remove all these things – I think it would be easier to adjust classes especially for them and then we could also participate there. (Interview 1)

Another issue was when people with disabilities should use the fitness centre. Some of the participants assumed that people with disabilities would use the fitness centre in daytime, and therefore not take up the more desirable times after normal working hours from 4 to 8 pm.

A sense of community was important for the participants, and they said that they wanted people with disabilities to be part of that as well. They valued diversity and that everyone should feel welcome, regardless of age, background, or social class. But at the same time, participants thought it much easier to be tolerant and inclusive towards people with physical disabilities in contrast to people with cognitive issues or mental disabilities making it difficult to follow the codex for ‘normal’ interpersonal behaviour. Birger explained:

I don’t know if it is wrong to call it for a social disability/handicap, but if you do not have boundaries like most other people, you could bother other users in the fitness centre, that would be a problem. (Interview 3)

Being part of a voluntary-based community, it is important to help each other and create a culture where all people take care of the place and clear up after oneself. Participants valued this kind of atmosphere where members helped each other during exercise. This also involved helping people with disabilities, but only to a certain extent, as participants did not want to be obligated to help or be delayed in their own exercises. Maya reflected:

I don’t mind sharing the fitness centre with disabled people, but on the other hand I would be annoyed if I went to exercise and ended up behind a wheelchair user who takes forever to transfer between the fitness machines. It is not nice to say I know, but I would be annoyed. (Interview 1)

#### Interaction with users with disabilities

Finally, several participants stated that they had some fear of interacting with people with disabilities because they were afraid to do something wrong or be misinterpreted. They wanted everybody to feel comfortable but felt insecure regarding how to behave so that they did not unintentionally offend. Josefine elaborated:

Either you have reluctance to deal with people with disabilities or you want to help, but they don’t need your help and react with disappointment if you ask. It is problematic, should you ask, or shouldn’t you? Do you look at them or should you not when you yourself are non-disabled and they are disabled? You need to take these problems into account, so everybody feels comfortable, and you don’t get snapped at and refuse to engage or talk to this [disabled] person again. (Interview 2)

Participants believed that no one should be offended, disappointed, insulted or snapped at, so that people with disabilities had the experience of dignity and pride. In general, the participants were very engaged in how to do things right, be respectful and treat people with disabilities as everyone else. Ib summed up:

It sounds like a cliché, but you have to respect them as they are, I can’t explain it in any other way. (Interview 3)

Some participants felt that they lacked social competences on how to do interact in practice because of their limited relations with people with disabilities in daily life. Henning shared his thoughts on how to handle specific situations:

I would just say: ‘You just give me a sign if you feel in need of help’. Then you have not directly asked, and they do not have to say no. Then they know that if they have a need for help, they can get it. (Interview 2)

The above section highlights that participants may have good intentions regarding sharing a space with members with disabilities, but it is apparent through many comments that there are inherent ableist perceptions and biases held by non-disabled members. These perceptions shed a light on the various disabling encounters that must be addressed during the conception of an inclusive fitness centre to avoid the pitfalls of early research.

## Discussion

Participants expressed opinions about the ‘right’ settings for non-profit club-based fitness centres with room for comfort, inclusion, and diversity, and how the ideal inclusive fitness centres should be to include people with disabilities, but ableist perceptions were apparent throughout. In this section, by means of four discussion points, we discuss how these expectations and suggestions may be operationalised with reference to existing knowledge on inclusive fitness centres, and potential pitfalls regarding ableism and how this must be considered when designing and inclusive fitness space.

### Non-profit fitness centres compared with commercial fitness centres

Participants had certain expectations and ideas about the ideal fitness centre based on their experiences with commercial fitness centres and non-profit fitness centres.

In general, and in line with existing knowledge, participants stated several important issues when choosing a fitness centre, such as locations with easy access [32–34] clean and well-maintained settings with a variety of up-to-date equipment [35, 36] and a centre not too crowded [37], noisy or smelly [38]. When participants evaluated the settings, it all came down to how the space affected them; how it made them feel. These findings underline that people have different preferences [39, 40], and this can explain the booming fitness industry whereby the centres become more and more niche orientated. Our findings further highlight the importance of creating a welcoming and comfortable space.

The participants preferred low-cost memberships as previously reported [35, 41], and quickly calculated the price per expected visit when arguing expense. On the other hand, they also preferred equipment with high standards found in commercial centres, so this is a trade-off to be aware off. Room for socialising was much in demand in non-profit fitness clubs in contrast with commercial centres where places to meet before and after almost are non-existent. Good social relations and a sense of community were highlighted in several studies [42–44], but for the current participants, it differed because they prioritised social relations beyond training. Studies of regular fitness centre users in commercial centres also stressed friendship both inside and outside the fitness centre [45]. We found, however, that room for socialising and focus on social relations may attract a specific kind of user to the non-profit fitness centres instead of commercial fitness centres.

### Motivating atmosphere

The atmosphere in a fitness centre was very important for the participants and they kept returning to this topic, stressing an atmosphere and welcome and invite motivated their use. In that sense, a good atmosphere is prioritised over functionality which is notable as a good atmosphere has not been widely described as motivating in previous studies focusing on non-disabled people. Generally, studies concluded motivation related to improving body appearance and performance, reducing health issues or improving mental well-being [39, 46–50]. In only three studies the atmosphere was associated with feelings of being comfortable, valued and welcomed [34, 42, 51].

To further create a motivating atmosphere, participants highlighted the importance of fitting in and belonging, regardless of age, bodily appearance, clothes, or type of training preferences, which could be facilitated through verbal and non-verbal interactions with members and staff. Indeed, staff members play key role in creating a good atmosphere [52, 53], which may be the reason why participants requested rules for behaviour and staff to enforce them to avoid stigma and enhance pleasant experiences for everybody.

Regarding ensuring welcome and invitation, participants were concerned about newcomers’ lack of knowledge and confidence entering a fitness centre. For beginners, fitness equipment can be complicated, so guidance is needed on both what to do and how to do it right. These issues have not been well established previously, but is described in relation to older adults [43, 50]. Lack of skill and knowledge may be considered a barrier that needs further consideration if all new members with limited or no experience should be included in fitness centres as it is not only related to age. Staff and other fitness centre members can play a key role helping and introducing newcomers to the space, trainings, and equipment to ensure a welcoming, inviting atmosphere.

### Interactions with people with disabilities – lack of experience

Participants believed they were welcoming of people with disabilities in fitness centres and expressed a more positive attitude compared to other countries. For example, only three quarters of participants in a survey from the UK were open to taking part in sport or active recreation with people with disabilities [54]. The current participants were overtly openminded, but also foresaw many potential barriers for inclusion on a more interactive level, especially when including persons with intellectual disabilities, due to a lack of social codex which is supported by previous studies [55, 56]. This is further supported in our specific work in Denmark as early analysis of focus groups from persons with disabilities points to similar findings. For example, in a quote from one of the interviews, Maria described problems when interactions between persons with and without disabilities go wrong:

“Many things regarding people with disabilities are kind of shushed down and you should not ask as a non-disabled person. But it results in non-disabled people not knowing, - they are not mean, they just don’t know better. They don’t know how to do or how not to do in situations you are not familiar with, and then it gets awkward, and you might say things that are taken in differently than they were meant. I think it goes both ways.” (Interview 3a with a group of participants with physical disability)

This example is provided for context and an in-depth focus on people with physical disabilities is forthcoming.

While the participants in this study in general had a positive attitude, they expressed a distinct absence of interactions with people with disabilities; they simply lacked experiences from their daily life with this group of people. This might be due to the national sport organisation where leisure time sports activities are split in traditional sport and parasport which does not favour inclusion. If attitudes, however, predict behaviour then inclusive fitness centres have a good starting point supported by a global movement with more positive attitudes to people with disabilities [57]. Intergroup contact theory [58] describes how direct contact between groups work in changing attitudes and reducing prejudice. This theory has been used in disability inclusive efforts previously including within schools [59], university [60] and the workplace [61], and may have considerable impact within a fitness centre. However, this is the case only assuming that positive attitudes towards people with disabilities is not just a consequence of politically correctness but reflecting their actual attitude. Yet, while participants perceived they had positive attitudes to people with physical disabilities, in general, they struggled with defining and exemplifying the group of people with physical disabilities. They found it difficult not to stigmatise when talking about ‘the others’ and ‘us normals’. Unfortunately this common way to portray people with disabilities as ‘other’ and not an integral part of the ‘normal’ world may be a barrier for social inclusion [57]. The non-disabled participants tried to omit these expressions, but they lack concepts and terminology to express themselves otherwise, highlighting unconscious ableist attitudes.

### Ableism: what is normal?

The participants welcomed inclusive fitness centres but did not pay much attention to how fitness centres could be inclusive, except for mentioning the obvious; the need for accessible environment and adaptive fitness equipment, but this is only one element of inclusion and will primarily be solved by the fitness centre and not by the participants themselves. The participants were not aware of their own implicit bias and role in exclusion regarding different ableist aspects of prejudice and ignorance, which can be a vital barrier for inclusion as it can lead to direct psycho-emotional disablism. For example, within the context of this study, participants expressed the importance of helping others to create a positive atmosphere but stated that persons with a disability requiring regular assistance may become annoying. Further, participants discussed the importance of supporting people with disabilities, but were concerned about time, resources and staff being taken away at the expense of non-disabled users. These ableist examples may negate an inclusive effort and result in persons with disabilities experiencing direct psycho-emotional disablism.

The issue about direct psycho-emotional disablism is further supported by preliminary analysis from our focus groups with participants with disabilities about their perspectives for inclusive fitness centres. One quotation from Caroline underlined her experiences of ableism from non-disabled persons:

“Maybe it is also the fear of actually living up to some of the prejudices [about persons with disabilities] you feel that [non-disabled] people are looking at you and if you ask for help you feel the look even stronger.” (Interview 1a with a group of participants with physical disability)

In the literature direct psycho-emotional disablism is both related to other fitness centre users, staff members, and management (all arguably influenced by ableist perceptions) [13, 62]. This narrow perspective is what Anderson et al. describe as an ableist-environment being exclusive towards people with disabilities [63]. The non-disabled participants in this study are, not surprisingly, viewing inclusive fitness centres through the lens of their perspective and they mention several situations where they imagine irritation with people with disabilities. Chouinard would characterise this as ableism of ideas, practices, institutions and social relations that presume able-bodiedness, and by doing so, construct persons with disabilities as marginalized, oppressed and largely invisible ‘others’ [64]. This is stigma that should be avoided but might be difficult to counter unless the perspective of both fitness users with and without disabilities are represented and included when establishing and running the new inclusive fitness centres. In that way, ‘normal’ is not defined by the non-disabled group of people with an (unconscious) ableist perspective but as the variety of both people with and without disabilities using the fitness centres.

### Limitations and future directions

To improve naturalistic generalisability, we strove for maximal variation within the group of adult non-disabled potential participants in the upcoming inclusive fitness centres, which we successfully achieved in terms of gender, age, and fitness centre experience. The participants were geographically recruited in 3 different parts of Denmark and are anticipated to be representative for all of Denmark. However, they are not representative for commercial fitness centre users, which is arguably a space with heightened ableism that excludes persons with disabilities [13]. This is a market that continues to see growth, thus if ableism is not challenged in this space as well, exclusion of people with disabilities may become even more of an accepted norm. Research focusing on inclusive efforts to resist ableism and disablism in other fitness spaces, such as commercial centres, are essential in order to stop the continued acceptance and normality of ableist practices in the fitness domain.

This article focused on the perspective of the non-disabled fitness users of the coming inclusive fitness centres, but of course the perspective from fitness users with physical disabilities were very important as well. Their perspective will be presented in an upcoming publication, based on three comparable focus group interviews. Studies show that the perspective of fitness users with disabilities is underrepresented in the scientific literature [65]. However, barriers for people with disabilities is reported when wanting to participate in gym-based exercising e.g. lack of accessibility, lack of social support, oppressive attitudes within gyms [7, 65], and further, instructors/staff have a key role in promoting inclusiveness or the opposite [66, 67] in fitness centres. Less has been written about the overt and unconscious ableism that must also be addressed to craft inclusive fitness spaces. While we did focus on ableism as a lens in our work, more much be done to explore the foundations, influences and strategies to dismantle ableism not only in the fitness domain, but wider society. A further limitation of the study is that the ‘Fitness for All’ initiative may only be applicable in Denmark and similar cultures as disability is so socially, culturally, and politically influenced. We encourage other countries to address the ableism, attitudes, and socio-cultural influences that shape attitudes and discrimination of people with disabilities within their own specific cultures and share ideas for interventions to create more inclusive fitness spaces. In this way, we can create a global inclusive movement such that there is better understandings and support of disability, culture and potential contributions and collaborations that may be made across countries.

## Conclusion

This is one of the first papers to explore the perceptions of inclusive fitness centres within Denmark, thereby adding essential knowledge to the literature. This paper's aim was shaped by two key questions; (1) to identify the ideal fitness space from the perception of non-disabled users and (2) to explore their dis/ableist attitudes related to the future inclusive fitness centres. First of all, participants pinpointed the importance of a place with a good atmosphere—a place that made them feel welcome and gave them a feeling of belonging. The participants mirrored themselves in relation to other users and aspects, like body ideals, gender, age, exercise preferences, and furthermore social relations were found important when they consider whether they fit in or not. Therefore, it is important that fitness centres not only focus on location and advanced fitness equipment, but also how to create the right atmosphere.

Participants welcomed people with disabilities and wanted them to feel included in the fitness community, but they predicted challenges for the future inclusive fitness centres and expressed unconscious prejudices. This underlines that accessibility (indirect psycho-emotional disablism) is not the only barrier for inclusion, since social skills, ableism, ignorance, and preconceptions can be important barriers too (direct psycho-emotional disablism). Inclusive fitness centres must address this so the definition of ‘normal’ is not only defined by the non-disabled group with an unconscious ableist perspective. This could be adjusted, e.g., by having staff members who are good role models to uphold policies and rules, by having both fitness users with and without disabilities joining the fitness centre and even have fitness users with disabilities as a part of the staff to make a greater impact. We need, however, to research the perceptions of people with disabilities regarding inclusive fitness centres and this will be presented in a forthcoming publication.

## Supplementary Information

**Additional file 1.**

## Data Availability

The datasets generated and/or analysed during the current study are not publicly available due to confidentiality of the participants but are available from the corresponding author on reasonable request.

## Abbreviations

IFC: the inclusive fitness coalition
IFI: the inclusive fitness initiative
STC: systematic text condensation
