# Supplementary material for: Fitness for all: how do non-disabled people respond to inclusive fitness centres?
Source: BMC Sports Sci Med Rehabil. 2021 Jul 30;13:81. doi: 10.1186/s13102-021-00303-2 (PMC8325230; doi:10.1186/s13102-021-00303-2)
Supplement: Supplementary file 1 — Additional file 1. [file 13102_2021_303_MOESM1_ESM.docx]

Interview guide – translation from Danish to English

| **Themes** | **Cues/subthemes** | **Questions** |
| --- | --- | --- |
| Introduction | participation  motivation | What are your experiences with fitness centres?  Can you share some examples with us?  (Both good and bad experiences)  Why do you use the fitness centre? /  What would it take for you to use it? |
| 1.  The physical surroundings/ accessibility | Transportation / location  Reception area  Clear floor space  Ramps/stairs/lifts  Locker room/bath/toilets  Fitness area  Classes/studio  Fitness machines/exercise equipment  Mirrors  psycho-emotional disablism   - indirect (structural barriers)   Economy? | What is good accessibility to you?  Where do you experience problems?  What is important and why?  What is good interior design in a fitness centre?  Any examples and experiences? |
| 2.  Activities/  usability | Individual exercising  Classes  Events  Supervision  Social activities  Staff? | How do you use the fitness centre (both in the past, present and future)?  What do you do when you are there?  What kind of activities are important?  What does it take to makes a fitness centre usable? |
| 3.  Atmosphere/  feeling | Relations to other users and staff  Preconceptions/stigmatising  Disability vs. non-disability  psycho-emotional disablism   - direct (looks, words or actions)   Communication | Give some examples of unwritten rules/gym etiquette?  Dos and don’ts…  What makes it comfortable to be in a fitness centre?  Any experiences?  Do you have any experiences with preconceptions/stigmatising in fitness centres? explain  How do we make a successful inclusive fitness centre for both people with and without physical disability? Pros and cons?  What should we be aware of and what are the challenges?  How are you being treated in the fitness centre?  Was your participation effected?  How did you handle the situation?  Could the physical environment remedy or prevent this? |
| Recapitulation | ”The ideal fitness centre”  Sum up | What is the most important key points to pass on into the Fitness for all-campaign?  What is the most important things in a (coming) fitness centre?  Other comments? |
